# Roles of Melatonin in Goat Hair Follicle Stem Cell Proliferation and Pluripotency Through Regulating the Wnt Signaling Pathway

**DOI:** 10.3389/fcell.2021.686805

**Published:** 2021-06-04

**Authors:** Weidong Zhang, Niu Wang, Tongtong Zhang, Meng Wang, Wei Ge, Xin Wang

**Affiliations:** ^1^College of Animal Science and Technology, Northwest A&F University, Yangling, China; ^2^College of Life Sciences, Qingdao Agricultural University, Qingdao, China

**Keywords:** melatonin, cashmere, hair follicle stem cells, Wnt pathway, CTNNB1

## Abstract

Emerging studies show that melatonin promotes cashmere development through hypodermic implantation. However, the impact and underlying mechanisms are currently unknown. *In vitro* study has previously demonstrated that melatonin induces cashmere growth by regulating the proliferation of goat secondary hair follicle stem cells (gsHFSCs), but there is limited information concerning the effects of melatonin on cell pluripotency. It is also known that Wnt signaling may actively participate in regulating cell proliferation and stem cell pluripotency. Therefore, in the current investigation, goat hair follicle stem cells were exposed to multiple concentrations of melatonin and different culture times to reveal the relationship between melatonin and the activation of Wnt signaling. A proportionally high Catenin beta-1 (CTNNB1) response was induced by 500 ng/L of melatonin, but it was then suppressed with the dosages over 1,000 ng/L. Greater amounts of CTNNB1 entered the cell nuclei by extending the exposure time to 72 h, which activated transcription factor 4/lymphoid enhancer-binding factor 1 and promoted the expression of the proliferation-related genes *C-MYC*, *C-JUN*, and *CYCLIND1*. Moreover, nuclear receptor ROR-alpha (RORα) and bone morphogenetic protein 4 (BMP4) were employed to analyze the underlying mechanism. RORα presented a sluggish concentration/time-dependent rise, but BMP4 was increased dramatically by melatonin exposure, which revealed that melatonin might participate in regulating the pluripotency of hair follicle stem cells. Interestingly, NOGGIN, which is a BMP antagonist and highly relevant to cell stemness, was also stimulated by melatonin. These findings demonstrated that melatonin exposure and/or NOGGIN overexpression in hair follicle stem cells might promote the expression of pluripotency markers Homeobox protein NANOG, Organic cation/carnitine transporter 4, and Hematopoietic progenitor cell antigen CD34. Our findings here provided a comprehensive view of Wnt signaling in melatonin stimulated cells and melatonin mediated stemness of gsHFSCs by regulating NOGGIN, which demonstrates a regulatory mechanism of melatonin enhancement on the growth of cashmere.

## Introduction

Melatonin (*N*-acetyl-5-methoxytryptamine), a conserved pineal gland secretory product, which has been documented for more than 60 years is known to regulate many physiological events, such as circadian rhythmicity, seasonal adaptation, and reproductive changes (Johnston and Skene, 2015; Slominski et al., 2018). In domestic animals, the secretion of melatonin is regulated by natural daylight showing a circadian rhythm and higher serum concentration in seasons with a shorter day length (Nozaki et al., 1990; Buchanan et al., 1992; Alila-Johansson et al., 2001).

Cashmere is a type of amyelinic fiber produced from secondary hair follicles (SHFs) in Cashmere goats. The growth of cashmere is photoperiod-dependent, and shows seasonal changes in many goat breeds (Zhang et al., 2019b). Each year, cashmere starts growing in late-summer, halts growth in mid-winter, and naturally sheds in spring. These cyclical changes shift through three periods of cell activity: anagen, catagen, and telogen (Zhang et al., 2014). Similar periodic processes of correlation have been noted between hair follicle growth and serum melatonin levels. Higher levels of melatonin are secreted in early anagen, which suggests that melatonin probably influences the cashmere growth process (Emesih et al., 1993; Chong et al., 2019; Zhang et al., 2019a). Of particular interest, melatonin exhibits characteristic biological functions in regulating goat hair growth (Fu et al., 2014; Li et al., 2019; Yang et al., 2019b). Following subcutaneous melatonin implantation, hair follicle development in newborn goats is significantly increased (Yang et al., 2019a). Melatonin boosts cashmere production by altering the hair growth cycle and structure (O’Neill et al., 1992; Duan et al., 2015; Ge et al., 2018; Feng and Gun, 2021). It has also been shown that, *in vitro*, melatonin enhances the elongation of cashmere hair shafts by promoting the proliferation of hair follicle stem cells (Ge et al., 2018). However, there is limited information concerning the mechanism of melatonin action on hair growth.

The Wnt pathway is essential to the initiation of hair growth (Andl et al., 2002; Zhang et al., 2009; Feng and Gun, 2021), and transmits signals among many types of stem cells during the formation of hair placodes (Zhang et al., 2009). Catenin beta-1 (CTNNB1), a downstream signal molecule in the Wnt pathway, is an essential factor in deciding hair follicle stem cell fate and mediating the formation of follicular keratinocytes (Huelsken et al., 2001). Following a Wnt signal, CTNNB1 translocates to the nucleus, triggers the CTNNB1/TCF enhancer factor transcriptional machinery, and finally upregulates downstream factors (Ishitani et al., 2003). Transcription factor 4 (TCF4) always works as an intranuclear member of CTNNB1/TCF enhancer factors which activate or inhibit the transcription of target genes (Omer et al., 1999). It has been reported that TCF4 regulates the proliferation and secretory activity of dermal papilla cells by regulating downstream gene expression (Xiong et al., 2014). Complex assemblies of CTNNB1/lymphoid enhancer-binding factor 1 (LEF1) also activate target transcription genes and act as promoters of hair follicle stem cell cornification (Driskell et al., 2007). Insufficient LEF1 leads to a reduction of body hair and the loss of whiskers (Van Genderen et al., 1994). Our previous study found that CTNNB1 was differentially expressed in melatonin exposed goat secondary hair follicle stem cells (gsHFSCs) compared with the control group, which revealed that melatonin is involved in regulating the state of gsHFSCs through the Wnt pathway (Ge et al., 2018). However, the regulatory mechanism of melatonin needed to be described more clearly.

In the current study, gsHFSCs were exposed to different concentrations and time periods of melatonin, and an optimum melatonin exposure concentration and time were determined by comparing the expression of CTNNB1 in the cells. Subsequently, TCF4 and LEF1—the downstream factors of *C-MYC*, *C-JUN*, and G1/S-specific cyclin-D1 (*CYCLIND1*)—were detected to further determine the roles of melatonin in the Wnt activation of gsHFSCs. Moreover, the cell growth and differentiation related factors of bone morphogenetic protein 4 (BMP4)/NOGGIN and the stemness related factor homeobox protein NANOG (NANOG), organic cation/carnitine transporter 4 (OCT4), and hematopoietic progenitor cell antigen CD34 (CD34) were also detected in gsHFSCs after melatonin stimulation. These findings broaden our understanding of the mechanisms involved in melatonin-dependent goat cashmere growth.

## Materials and Methods

### Isolation and Cultivation of gsHFSCs

The method for isolating gsHFSCs was similar to that previously reported (Ge et al., 2018). Briefly, goat secondary hair follicles were separated with ophthalmic forceps from newly collected fresh goat skin samples of body side, then the hair bulb regions of the separated follicles were dissected and trypsinized using 0.25% trypsin-EDTA solutions. The gsHFSCs were cultured in adherent culture dishes with DMEM/F12 media containing penicillin (100 U/mL), streptomycin (100 mg/mL), 20 ng/mL epidermal growth factor, and 40 ng/mL recombinant human FGF-basic. After authentication of cells using the hair follicle stem cell markers ITGB1, CD34, K15, and non-hair follicle marker K10, different dosages of melatonin were added to cell culture media to explore the optimum concentration (125, 250, 500, 1,000, 2,000, and 5,000 ng/L) and exposure time (12, 24, 36, 48, 60, 72, and 84 h).

### Immunofluorescence Analysis

Cells grown on coverslips were fixed with 4% paraformaldehyde for 15 min. They were then permeabilized in 0.5% Triton X-100 PBS solution for 15 min, and incubated in block solution (PBS with 0.5% triton X-100 and 10% goat serum) for >30 min at room temperature (RT). After blocking, the cells were incubated overnight at 4°C with primary antibodies against CTNNB1, LEF1, TCF4, C-MYC, C-JUN, CYCLIND1, CDK6, nuclear receptor ROR-alpha (RORα), BMP4, NOGGIN, NANOG, OCT4, and CD34 (shown in Table 1). Then, after rewarming to RT, the IgCY3/FITC-conjugated secondary antibodies (Abcam, ab150078 or ab150073) were incubated for 1 h, and Hoechst 33342 was used to stain the nuclei. Finally, slides were examined under an Olympus fluorescence microscope imaging system, and the fluorescence intensity data was analyzed with ImageJ.

**TABLE 1:** Information of primers for real-time quantitative PCR.

Gene symbol	Whole gene name	GenBank	Primer sequence (5′-3′)	Prodution size (bp)	Tm
		**Accession no.**			
Beta-ACTIN	Actin beta	NM_001314342.1	5′- GGAATCGTCCGTGACATCAA -3′	107	58
			5′- AGCTCGTAGCTCTTCTCCA -3′		
BMP4	Bone morphogenetic protein 4	XM_013967192.2	5′- AGATGGTAGTGGAGGGATGT -3′	111	62
			5′- GTGAGTAGTGGATGGGATGTG -3′		
CDK6	Cyclin-dependent kinase 6	XM_018047425.1	5′- CTCCAGTCCCACAATCCTAAC -3	120	62
			5′- CGTCTCAGTGATGGAGAAGAAC -3		
CTNNB1	Catenin beta-1	XM_018066894.1	5′- GAGGACAAGCCACAGGATTAT -3′	101	58
			5′- CCAAGATCAGCGGTCTCATT -3′		
C-JUN	AP-1 transcription factor subunit JUN	XM_018044742.1	5′-CGAAGTGACGGACTGTTCTATG -3′	134	62
			5′-TCATGCTCTGCTTCAGAATCTT -3′		
C-MYC	Myelocytomatosis oncogene Tag	XM_018058563.1	5′-CAGAGGAGAAACGAGCTGAAA -3	130	62
			5′-CTTGGACCGACAGGATGTATG -3		
CYCLIND1	Cyclin D1 (CCND1)	XM_018043271.1	5′-GCAGTCTTAGGCATCCTGTAT -3	131	58
			5′-CCTAGCCGAGAGGTTACATTA- 3		
LEF1	Lymphoid enhancer binding factor 1	NM_001285746.1	5′- CAGGTGGTGTTGGACAGATAA -3′	95	62
			5′- ATGAGGGATGCCAGTTGTG -3′		
RORα	RAR related orphan receptor alpha	NM_001285652.1	5′- CTTTCACCAACGGAGAGACTT -3′	124	62
			5′- GTTATCTGCTGGAGCTCTTCTC -3′		
TCF4	Transcription factor 4	XM_018039407.1	5′- CACTTTCCCTAGCTCCTTCTTC -3′	136	62
			5′- GTAGCTGCTAGACTGTGGAATG -3′		
NOGGIN	Noggin	XM_013971792.2	5′- GCCAGCACTATCTCCACATC -3′	110	62
			5′- CTCGTTCAGATCCTTCTCCTTG -3′		

### Real-Time Quantitative PCR Analysis

Total RNA was extracted from the cells exposed to melatonin using Trizol and dispersed in RNase-free water. Then the first cDNA strands were obtained using a PrimeScript^TM^ RT reagent kit with gDNA Eraser (Takara, Cat# RR047A). Gene-specific primers for amplification were designed using Primer 5 software and shown in Table 2. Real-time quantitative PCR (RT-qPCR) was performed with a Roche LightCycler^®^ 96 System using TB Green Premix Ex Taq^TM^ II (Takara, Cat#RR820A). The RT-qPCR protocol was as follows: 95°C for 3 min, followed by 45 cycles of 95°C for 15 s and Tm value for 30 s.

**TABLE 2:** List of antibodies used for Immunofluorescence analysis and western blot.

Whole name	Name of antibody	Manufacturer	Cat No.
Actin beta	Anti-ACTB rabbit polyclonal antibody	Sangon Biotech	D110001
Bone morphogenetic protein 4	Anti-BMP4 rabbit polyclonal antibody	Sangon Biotech	D120315
Catenin beta 1	Anti-CTNNB1 rabbit polyclonal antibody	Sangon Biotech	D260137
Cyclin-D1	Anti-CCND1 rabbit polyclonal antibody	Sangon Biotech	D120509
Cyclin-dependent kinase 6	Anti-CDK6 rabbit polyclonal antibody	Sangon Biotech	D120398
Glyceraldehyde-3-phosphate dehydrogenase	Anti-GAPDH rabbit polyclonal antibody	Sangon Biotech	D110016
Hematopoietic progenitor cell antigen CD34	Anti-CD34 rabbit polyclonal antibody	Sangon Biotech	D263155
Jun proto-oncogene	Anti-JUN rabbit polyclonal antibody	Sangon Biotech	D220068
Lymphoid enhancer binding factor 1	Anti-LEF1 rabbit polyclonal antibody	Cell signaling	2286
Myelocytomatosis oncogene Tag	Anti-c-Myc Tag rabbit polyclonal antibody	Sangon Biotech	D110006
Noggin Organic cation/carnitine transporter 4	Anti-NOG rabbit polyclonal antibody Recombinant Anti-Oct4 antibody	Sangon Biotech abcam	D263592 ab200834
RAR related orphan receptor alpha	Anti-RORA rabbit ployclonal antibody	Sangon Biotech	D162141
Transcription factor 4	Anti-TCF4 rabbit polyclonal antibody	Sangon Biotech	D154040

### Western Blotting

Cell samples were lysed in RIPA buffer. Protein concentration was detected using a BCA kit (Solarbio, Cat#PC0020). Nuclear and cytoplasmic proteins of gsHFSCs were extracted using a Nuclear and Cytoplasmic Protein Extraction Kit (Sangon Biotech, Cat#C510001). GAPDH and β-Actin were used as the control. Aliquots of 50 μL of antigen per sample were loaded onto 8% SDS polyacrylamide electrophoresis gels. Then the gels were transferred to a polyvinylidene fluoride (PVDF) membrane at 300 mA for 1.5 h, at 4°C. Subsequently, the membranes were blocked with 5% BSA for 1 h at RT. The membranes were incubated with primary antibodies diluted at 1:500 in 0.1% Tween-20 in TBS (TBST) with 1% BSA overnight at 4°C. The next day, the blots were incubated with HRP-labeled secondary goat anti-rabbit or donkey anti-rabbit Ab for 1 h at RT and imaged after chromogenic reaction.

### MTT Assay

The gsHFSCs were seeded in 96-well plates at 2 × 10^3^ cells/well and cultured for 72 h after melatonin treatments. Then, 50 μL MTT solution [3-(4, 5-dimethylthiazol-2-yl)-2, 5-diphenyltetrazolium bromide, 5 mg/mL] was added to each well and the cells were incubated at 37°C for 4 h in the dark. To each solution, 100 μL of DMSO was added to dissolve the precipitate and blend in the dark at RT for 15 min. Absorbance values were determined at 490 nm and 630 nm using a multiwell microplate reader.

### Cell Cycle Detection

Trypsin digested gsHFSCs were fixed with cold 70% ethanol overnight. Cells were resuspended in 100 μL PBS after low speed centrifugation, then treated with 5 μL PI stain (Sangon, Cat# E607306) containing Rnase A (Beyotime, Cat# ST579) at 37°C for 30 min. The cells were immediately placed in a CytoFLEX flow cytometer and ∼20,000 cells were collected for analysis. Data were analyzed using a CytExpert system.

### Statistical Analysis

SPSS (v.20) was used for statistical analysis. Comparisons between groups were tested by one-way ANOVA and LSD tests. All groups were compared with each other for every parameter (mean ± SEM) and the differences were considered significant at ^∗^*p* < 0.05, ^∗∗^*p* < 0.01, and ^∗∗∗^*p* < 0.001.

## Results

### Melatonin Activated Wnt Signaling Pathway in gsHFSCs With an Increment of CTNNB1 Levels in Nuclei

**FIGURE 1:**
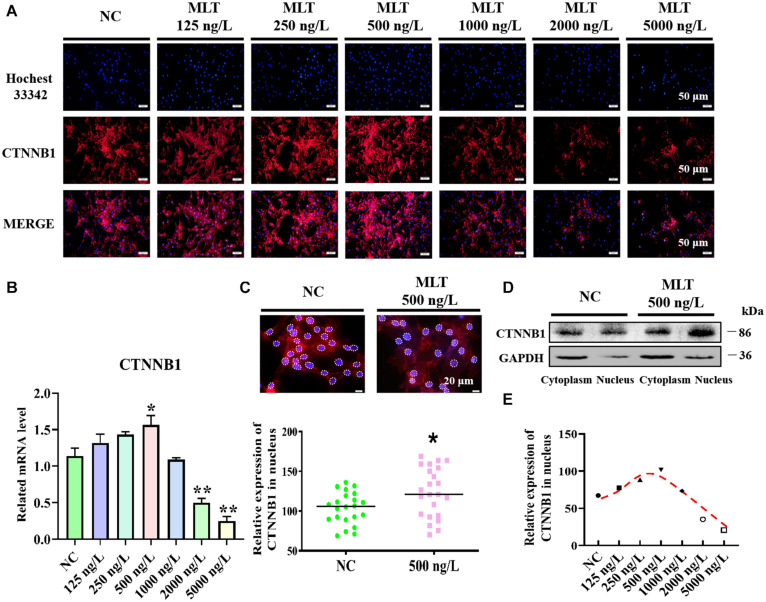
Melatonin activated Wnt signaling pathway in gsHFSCs. **(A)** CTNNB1 detected by immunofluorescence staining after stimulation with different concentrations of melatonin. Scale bar, 50 μm. **(B)** Relative mRNA level of *CTNNB1* by RT-qPCR; *p < 0.05, **p < 0.01. **(C)** CTNNB1 marked by immunofluorescence in gsHFSCs. Scale bar, 20 μm (white circles circumscribe the position of CTNNB1 in the nuclei), and relative immunofluorescence intensity of CTNNB1 in the nuclei; *p < 0.05. **(D)** CTNNB1 in nuclei/cytoplasm as detected by Western blotting. **(E)** Fluorescence density analysis of CTNNB1 in the nuclei of gsHFSCs.

Catenin beta-1, which is crucial for transmitting Wnt signals from the cytomembrane to the nucleus, was determined after 36 h of melatonin exposure. Immunofluorescence and RT-qPCR results suggested that melatonin mediated the activation of the Wnt pathway in gsHFSCs by modulating CTNNB1. The expression of CTNNB1 showed a concentration-related increase and then reduced with increasing doses of melatonin (Figure 1A). In particular, the expression of *CTNNB1* reached a maximum value at 500 ng/L, then abated with melatonin dosages over 1,000 ng/L (Figure 1B). In addition, more CTNNB1 became assembled in nuclei after melatonin exposure (Figure 1C), and melatonin significantly increased the CTNNB1 levels in nuclei (Figure 1D). Discrepant CTNNB1 was probed under different melatonin concentrations, which suggested that 250∼500 ng/L melatonin maintained high nuclear CTNNB1 levels in gsHFSCs (Figure 1E).

### Extended Melatonin Exposure Maintained the Continuous Activation of Wnt Signaling in gsHFSCs

**FIGURE 2:**
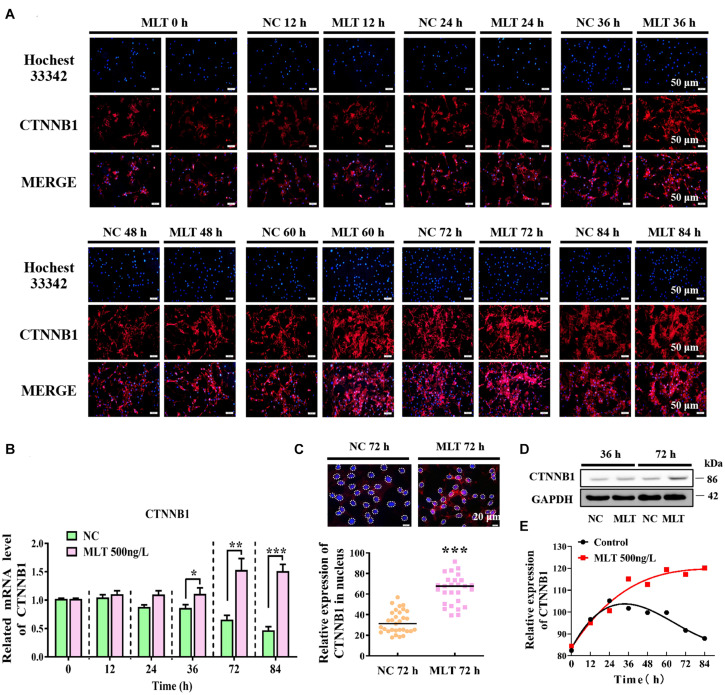
Melatonin maintained the continuous activation of Wnt signaling in gsHFSCs. **(A)** CTNNB1, as detected by immunofluorescence staining after stimulation by different periods of melatonin exposure. Scale bar, 50 μm. **(B)** Relative mRNA levels of *CTNNB1* (Control: green; melatonin: pink); *p < 0.05, **p < 0.01. **(C)** CTNNB1 marked by immunofluorescence in gsHFSCs. Scale bar, 20 μm (white circles circumscribe the position of CTNNB1 in the nuclei), and relative immunofluorescence intensity of CTNNB1 in nuclei; ***p < 0.001. **(D)** Protein levels of CTNNB1 between 36 and 72 h in treated gsHFSCs as shown by Western blotting. **(E)** Fluorescence density analysis of CTNNB1 in the nuclei of gsHFSCs after stimulation with different periods of melatonin exposure.

Since melatonin significantly increased the expression of CTNNB1, longer melatonin exposure times were implemented to explore whether the activation of Wnt signals could be maintained at 500 ng/L. The result showed that CTNNB1 was not affected at 24 h, but it was dramatically increased when exposure times were extended from 36 to 84 h (Figures 2A,B). At 72 h, the protein level of CTNNB1 significantly increased and more CTNNB1 entered the nuclei, which indicated that longer exposure time might induce the migration of CTNNB1 (Figures 2C,D). Subsequently, when prolonging the stimulation time of melatonin, CTNNB1 in nuclei was maintained at a high level compared with the control (Figure 2E).

### Melatonin Promoted the Expression of Wnt Downstream Factors in gsHFSCs

**FIGURE 3:**
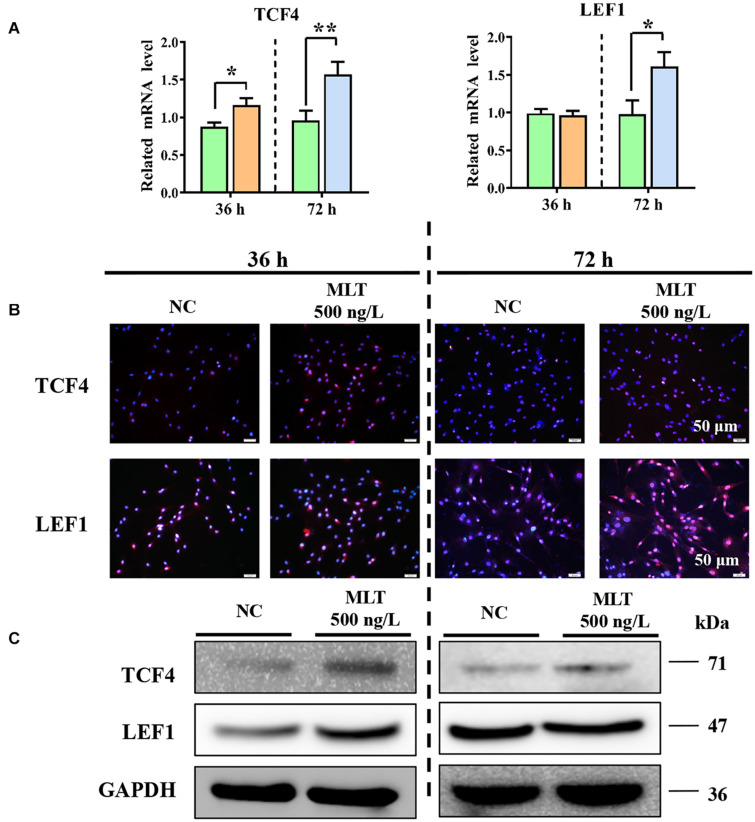
Melatonin promoted the expression of Wnt downstream factors in gsHFSCs. **(A)** Relative mRNA level of *TCF4* and *LEF1* as detected by RT-qPCR; **p < 0.01, *p < 0.05 (Control: green; 36 h melatonin: orange; 72 h melatonin: blue). **(B)** TCF4 and LEF1 tested by immunofluorescence staining. Scale bar, 50 μm. **(C)** TCF4 and LEF1 were compared between melatonin exposed and control cells using Western blotting.

Catenin beta-1 always works as part of the CTNNB1/TCF/LEF enhancer factor and mediates downstream factor transcription. Therefore, the levels of two important factors—TCF4 and LEF1—were determined to explore whether the complex binding was affected by melatonin. The expression of CTNNB1 was mediated at 36 h, but melatonin did not concurrently regulate *TCF4* or *LEF1* at mRNA level. The expression of *TCF4* was increased by melatonin exposure, but *LEF1* remained constant until 72 h (Figure 3A). Results from immunofluorescence and Western blotting also supported these findings (Figures 3B,C). Together, these data established that extending melatonin exposure time helped to maintain the activation of Wnt in gsHFSCs.

### Melatonin Promoted the Proliferation and Cell Cycle of gsHFSCs Through Stimulating Wnt Downstream Factors

**FIGURE 4:**
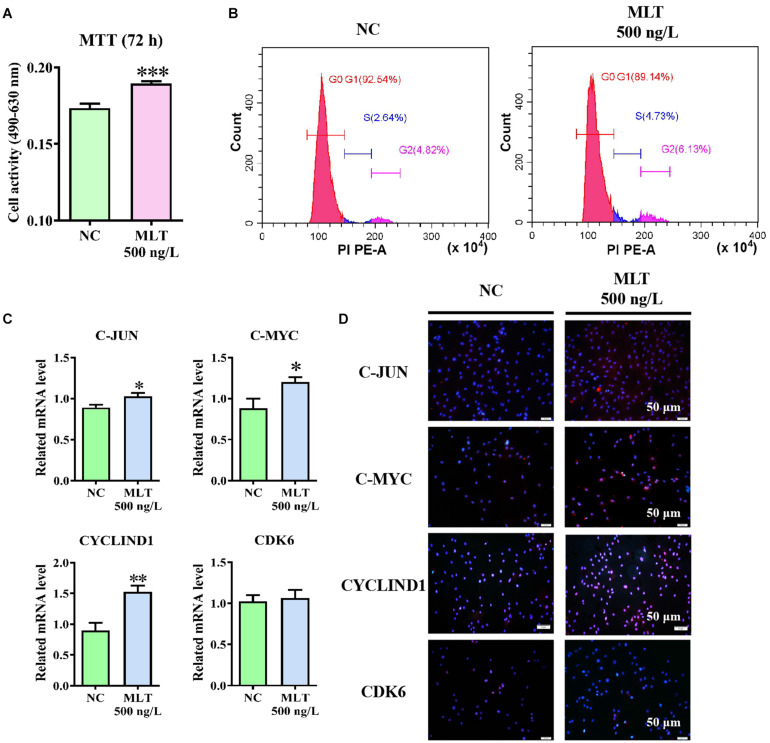
Melatonin promoted the proliferation and cell cycle of gsHFSCs by promoting Wnt downstream factor expression, ^∗∗∗^*p* < 0.001. **(A)** MTT analysis of melatonin on gsHFSCs proliferation following 72 h of exposure. **(B)** Cell cycle analysis of gsHFSCs after melatonin exposure at 72 h. **(C)** Relative mRNA level of *C-JUN*, C-MYC, *CYCLIND1*, and *CDK6* as detected by RT-qPCR after melatonin exposure for 72 h; ^∗∗^*p* < 0.01, ^∗^*p* < 0.05 (Control: green; 72 h melatonin: blue). **(D)** Proteins tested by immunofluorescence staining. Scale bar, 50 μm.

Cell viability assays were performed using the MTT assay to determine whether activation of the Wnt pathway was sufficient to promote the proliferation of gsHFSCs. The proliferation of gsHFSCs was increased after 72 h of melatonin exposure when compared with the control cells (Figure 4A). Cell cycle analysis further showed that the percentage of gsHFSCs in S phase and G2 phase were higher than those in unexposed cells (Figure 4B). Moreover, to explore the possible mechanism of melatonin in regulating the proliferation of gsHFSCs, the expression of downstream factors in the Wnt pathway and cell cycle-mediated factors, such as C-JUN, C-MYC, CYCLIND1, and CDK6 were analyzed by RT-qPCR and immunofluorescence staining after 72 h of melatonin exposure. The mRNA of *C-JUN* and *C-MYC* basically showed the same trend, but *CYCLIND1* was up-regulated significantly (Figure 4C). Higher protein levels of C-JUN, C-MYC, and CYCLIND1 were detected, but Cyclin-dependent kinase 6 (CDK6) remained constant after melatonin exposure (Figure 4D). These findings revealed that melatonin promoted the cell cycle by stimulating Wnt activation and might serve as a potential proliferation regulator in gsHFSCs.

### Melatonin Affected the Expression of RORα and Mediated the Equilibrium Between BMP4 and NOGGIN

**FIGURE 5:**
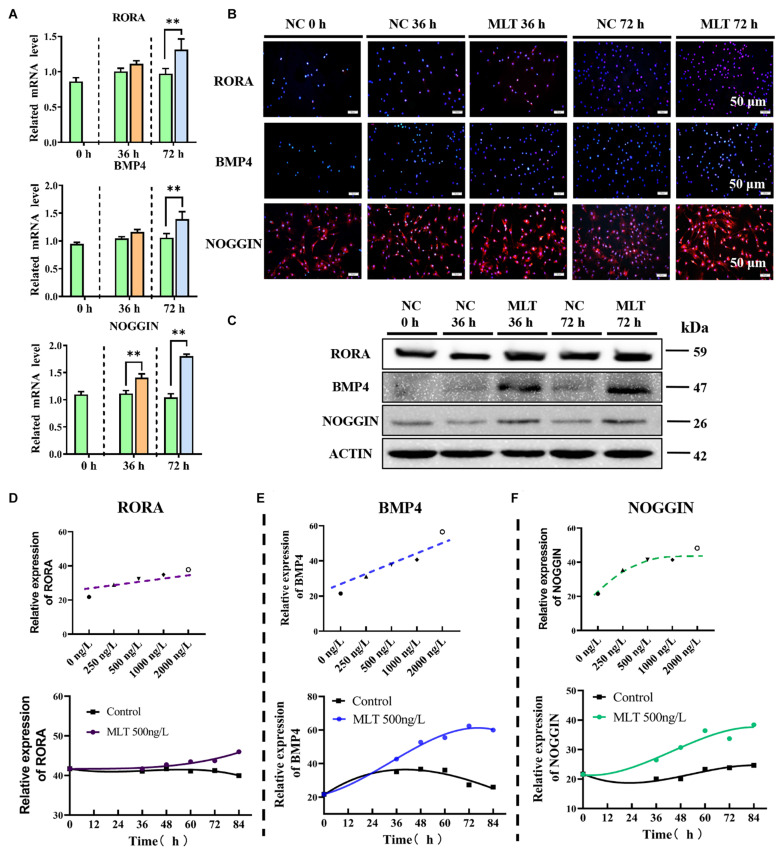
Melatonin mediated the expression of RORα, BMP4, and NOGGIN. **(A)** Relative mRNA levels of *ROR*α, *BMP4*, and *NOGGIN* in gsHFSCs before/after melatonin exposure (Control: green; 36 h melatonin: orange; 72 h melatonin: blue); ^∗∗^*p* < 0.01. **(B)** Protein levels of RORα, BMP4, and NOGGIN in gsHFSCs as revealed by immunofluorescence staining. Scale bar, 50 μm. **(C)** Protein levels of RORα, BMP4, and NOGGIN in gsHFSCs as shown by Western blotting. **(D)** Fluorescence intensity analysis of RORα in melatonin exposed gsHFSCs. **(E)** Fluorescence intensity analysis of BMP4 in melatonin exposed gsHFSCs. **(F)** Fluorescence intensity of NOGGIN in melatonin exposed gsHFSCs.

To explore the regulation of the mechanism of melatonin in gsHFSCs, the melatonin receptors MT1, MT2, and RORα, were measured in melatonin exposed gsHFSCs. The mRNA expressions of *MT1* and *MT2* were not detected in gsHFSCs (not shown). But the background expression level of *ROR*α was clearly observed (Figures 5A–C). A sluggish rise in RORα was observed in 36-h-melatonin-exposed gsHFSCs, but was not noticeable at 72 h (Figure 5A). The data from Western blotting and RT-qPCR supported a similar conclusion (Figures 5B,C). BMP4 and NOGGIN (a BMP antagonist) were highly related to the differentiation and pluripotency of gsHFSCs. Less BMP4 was detected in the control cells, but melatonin significantly increased its expression at 36 and 72 h (Figures 5A–C). Slightly different from BMP4, NOGGIN was constantly expressed in gsHFSC; a clear accumulation was noted when extending the melatonin exposure time, which suggested that melatonin always suppressed the signal of BMP4 by promoting the expression of NOGGIN (Figures 5A–C). Macroscopic details of RORα, BMP4, and NOGGIN were delineated by fluorescence intensity analysis under different melatonin conditions (Supplementary Figures 1A–C). RORα was not notably affected by an increase in melatonin dosage, but BMP4 and NOGGIN exhibited a dramatic improvement (Figures 5D–F). In addition, the expression of BMP4 and NOGGIN were time-dependent; they were increased by melatonin and always maintained a higher level than those in untreated gsHFSCs (Figures 5D–F). These data suggested that the pluripotency of gsHFSCs was regulated by the dynamic equilibrium of BMP4 and NOGGIN.

### Melatonin Meliorated the Pluripotency of gsHFSCs by Stimulating the Three Factors of NANOG, OCT4, and CD34

**FIGURE 6:**
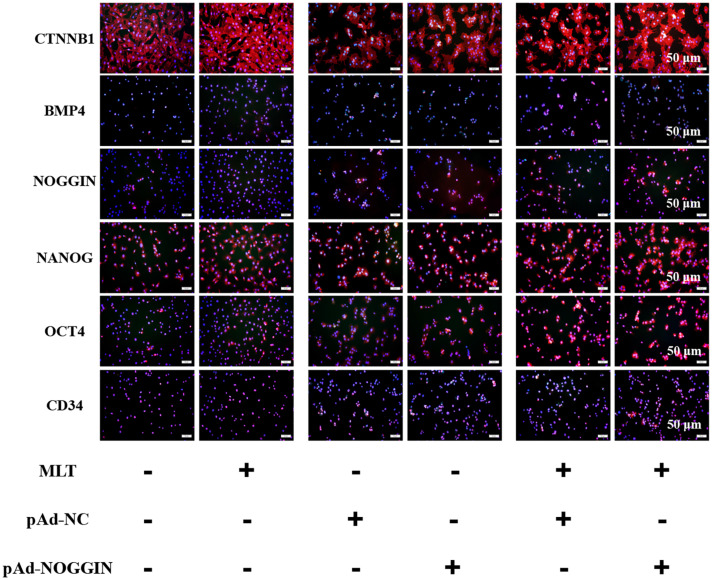
Protein levels of CTNNB1, BMP4, NOGGIN, NANOG, OCT4, and CD34 were detected after exposure to 500 ng/L melatonin with or without NOGGIN overexpression as shown by immunofluorescence staining at 72 h. Scale bar, 50 μm.

**FIGURE 7:**
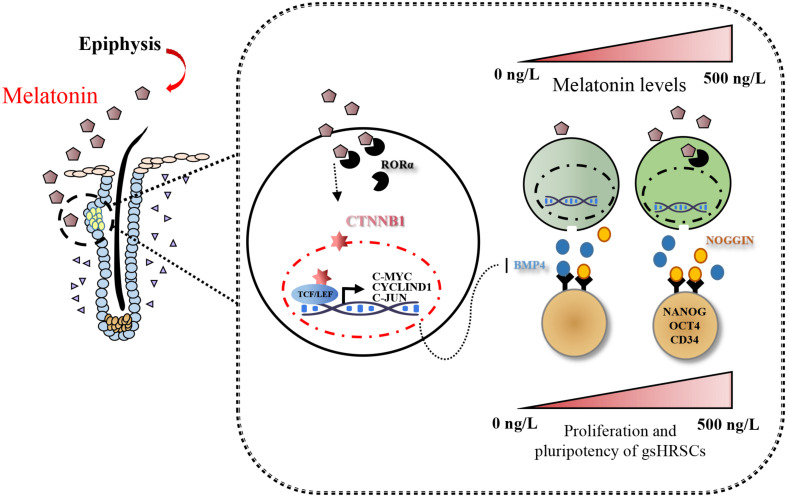
Model summarizing the main findings of this study.

For further analysis of the function of melatonin on hair follicle stem cell pluripotency, three stemness-related transcription factors—OCT4, NANOG, and CD34—were evaluated. NANOG and OCT4 were highly related to the self-renewal of stem cells. They were gradually increased in melatonin exposed cells, but the stemness marker CD34 was not affected (Figure 6). To clarify how melatonin mediated the pluripotency of gsHFSCs, NOGGIN was overexpressed in gsHFSCs. The results showed that CTNNB1 was significantly promoted, and the expression of NANOG and OCT4 revealed a similar rise to that in melatonin exposed cells. In the co-treatment group, the cumulation of CTNNB1 was observed clearly, but BMP4 expression was reduced. Furthermore, NANOG, OCT4, and CD34 were all maintained at a high level. Together, our data suggest that melatonin regulates gsHFSCs plurpotency by promoting the expression of NOGGIN (Figure 7).

## Discussion

The growth of cashmere is affected by many factors, such as photoperiod, nutritional aspects, feeding management, and environmental climate; these can be manipulated by farmers to control the process of cashmere growth and thus promote its production from Cashmere goats (Mcgregor, 1998; McGregor, 2009; Liu et al., 2016; Zhang et al., 2016; Yang et al., 2017). Among these factors, the control of photoperiod is considered to be the easiest and most cost-effective way to enhance cashmere production (Zhang et al., 2019b). Short duration sunlight accelerates the growth of cashmere, while long duration sunshine decelerates it (Chong et al., 2019). The mechanism of sunshine-mediated cashmere growth has, however, remained perplexing, although evidence suggests that melatonin might be a functional regulator in this process (Emesih et al., 1993). *In vivo* experiments have shown that cashmere growth can be increased by the implantation of subcutaneous melatonin (Sejian and Srivastava, 2010; Ghosh et al., 2014; Yang et al., 2019a, b). However, until recently, there has been little evidence to show that melatonin directly affects hair follicles. In 2018, an *in vitro* study revealed that specific hair follicle growth was observed in media containing 500 ng/L melatonin, which was considered to be the result of melatonin-mediated gsHFSCs proliferation (Ge et al., 2018).

Wnt signaling is closely related to cell fate determination, which is involved in stem cell proliferation, migration, and differentiation processes (Tang et al., 2019). So, this study mainly focused on CTNNB1 (a downstream signal molecule in the Wnt pathway), which is a key regulator in stem cell proliferation and self-renewal (Augustin, 2015; Veltri et al., 2018). We found that CTNNB1 mediated the physiological role of melatonin in a manner dependent on melatonin concentration and exposure time. CTNNB1 migrated into the nuclei and promoted the expression of the downstream genes. We observed that melatonin caused the discrepant expression of CTNNB1 when concentrations were >500 ng/L; this suggested that a threshold probably existed in the different response of melatonin functions (Shen et al., 2017; Knani et al., 2020). In cashmere growth, the proliferation of gsHFSCs is a prerequisite, but the stemness of gsHFSCs reduces with the extension of cell culture time (Zhang Y. et al., 2013). So, in the current study, we determined to detect whether melatonin could activate CTNNB1 in a sustainable manner. A stable activation of Wnt was discovered in melatonin exposed gsHFSCs, which could continue for over 72 h and help to maintain the proliferation and self-renewal of gsHFSCs. Wnt downstream factors TCF4 and/or LEF1, which are associated with dedifferentiation and acquisition of stemness properties (Merrill et al., 2001; Kim et al., 2020), became bound to CTNNB1 to co-regulate downstream gene expression (Dasgupta and Fuchs, 1999). Alteration of TCF4 and LEF1 in the current study also suggested that Wnt activation was the key to regulating melatonin-mediated cashmere growth. It has been shown that a high expression of TCF4 and LEF1 inhibits stem cell differentiation, which also plays important roles in skin regeneration and hair follicle formation (Aulicino et al., 2020; Yu N. et al., 2020). In this study, we demonstrated that melatonin might activate Wnt signals in gsHFSCs depending on the strength and duration of CTNNB1 signals, which has been detected in other melatonin exposed cells (Celso et al., 2004; Lowry et al., 2005; Silva-Vargas et al., 2005). Wnt signaling may activate the transcription of growth-related factors and mediate the cell proliferation of HFSCs (Lien et al., 2014; Xu et al., 2015). In this study, melatonin was also discovered to promote the expression of the Wnt downstream factors C-JUN, C-MYC, and CYCLIND1, which might mediate the proliferation of gsHFSCs. Melatonin propelled the cell cycle of gsHFSCs in a CYCLIND1-dependent way, and it was helpful to explore this mechanism in melatonin induced cell proliferation.

The intensity of different responses to melatonin could be related to the cell-line-specific pattern of melatonin cellular receptors and cytosolic protein expression (Zaminy et al., 2008). Furthermore, it is known that the activation of melatonin is mediated by G protein-coupled receptor melatonin receptor type 1, 2, and 3 (MT1, MT2, MT3, respectively, and directly) or the retinoid-related orphan nuclear receptor α (RZR/RORα, indirectly), and these receptors serve as attractive targets for a series of biological processes such as immunomodulation, endocrine regulation, and cancer formation (Foldes et al., 1992; Slominski et al., 2012). In our study, MT1 and MT2 were detected, but the background expression of RORα was discovered in gsHFSCs. RORα is reported to suppress the activation of Wnt (Lee et al., 2010). However, a contradictory result was obtained in the current study, which supported the existence of other mechanisms.

Nevertheless, we achieved a breakthrough in subsequent studies. Numerous research studies now show that the activation of Wnt is regulated by a series of signals, including BMP, FGF, and Notch pathways in HFSCs (Genander et al., 2014; Lien et al., 2014; Mesa et al., 2015; Binh et al., 2018; He et al., 2018). It is also well known that BMP4 regulates processes such as germ cell differentiation and the maintenance of self-renewing stem cells (Zhang J. et al., 2013; Kan et al., 2019). BMP4 is widely expressed in hair follicle stromal cells and the epidermis during embryonic hair follicle formation and the post-natal hair follicle cycle (Botchkarev et al., 2002). So, the expressions of BMP4 and NOGGIN were also examined here to measure the cell fate of gsHFSCs. The expression of BMP4 was notably increased in the presence of melatonin. The alteration of BMP4 is predictable here, because melatonin also mediates cell differentiation in series types of stem cells (Voeltzel et al., 2018; Zhang et al., 2018; Yu S. et al., 2020). NOGGIN works as an antagonistic protein in inhibiting the binding of BMP to its receptors (Chen et al., 2012; Xie et al., 2016); NOGGIN is also reported to maintain the pluripotency of stem cells (Chaturvedi et al., 2009; Díaz-Moreno et al., 2018). However, in the current study, NOGGIN showed an astonishing performance as a potential target of melatonin in gsHFSCs. We found that NOGGIN was overexpressed in gsHFSCs, and pluripotency markers were detected in gsHFSCs with/without melatonin exposure. The expression of NANOG was promoted under melatonin exposure and NOGGIN overexpressed gsHFSCs. In NOGGIN overexpressed gsHFSCs, melatonin magnified the acceleration of NANOG, OCT4, and CD34. NANOG participates in the transcription regulation of stem cells, but not in differentiated cells, and its ample expression meant that the stemness of cells was well maintained (Yamaguchi et al., 2005). Oct4 is known to be expressed in totipotent cells such as embryonic stem cells but its expression declines or disappears following cell differentiation (Yoo et al., 2011).

## Conclusion

Taken together, our findings provided a comprehensive view of the action of Wnt in melatonin stimulated cells; furthermore, melatonin mediated the stemness of gsHFSCs through its regulation of NOGGIN.

## Data Availability Statement

The original contributions presented in the study are included in the article/Supplementary Material, further inquiries can be directed to the corresponding author/s.

## Ethics Statement

The animal study was reviewed and approved by Laboratory Animals of the National Institutes of Health.

## Author Contributions

WZ and WG designed the research. WZ, NW, TZ, and MW performed the experiments and analyzed the data. WZ wrote the manuscript. XW has primary responsibility for the final content. All authors read and approved the final manuscript.

## Conflict of Interest

The authors declare that the research was conducted in the absence of any commercial or financial relationships that could be construed as a potential conflict of interest.

## Supplementary Material

Supplementary Figure 1 Protein levels of RORA **(A)**, BMP4 **(B)**, NOGGIN **(C)** were detected after exposed melatonin by immunofluorescence staining. Scale bar, 50 μm.

## Abbreviations

CTNNB1: Catenin beta-1
TCF4: Transcription factor 4
LEF1: Lymphoid enhancer-binding factor 1
RORα: nuclear receptor ROR-alpha
BMP4: Bone morphogenetic protein 4
NANOG: Homeobox protein NANOG
OCT4: Organic cation/carnitine transporter 4
CD34: Hematopoietic progenitor cell antigen CD34.
