# Supplementary figures and images for: Roles of Melatonin in Goat Hair Follicle Stem Cell Proliferation and Pluripotency Through Regulating the Wnt Signaling Pathway
Source: Front Cell Dev Biol. 2021 Jun 4;9:686805. doi: 10.3389/fcell.2021.686805 (PMC8212062; doi:10.3389/fcell.2021.686805)

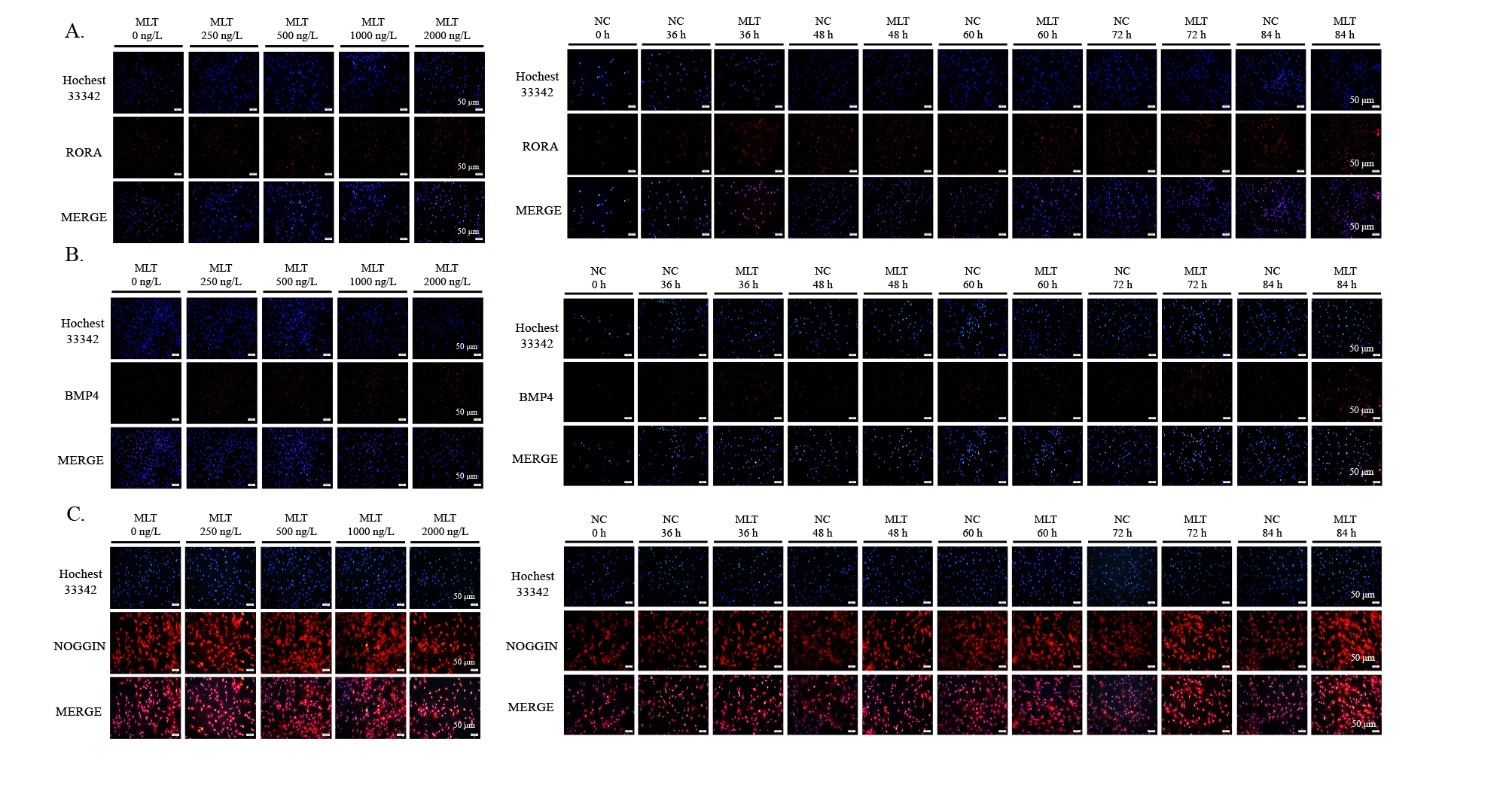

Supplement: Supplementary Figure 1 — Protein levels of RORA (A), BMP4 (B), NOGGIN (C) were detected after exposed melatonin by immunofluorescence staining. Scale bar, 50 μm. [file Image_1.TIF]
